# Themes and Theories Revisited: Perspectives on Processes in Family–Peer Relationships

**DOI:** 10.3390/children8060507

**Published:** 2021-06-15

**Authors:** Gary W. Ladd, Ross D. Parke

**Affiliations:** 1 Department of Psychology, Sanford School of Social and Family Dynamics, Arizona State University, Tempe, AZ 85287, USA; 2 Department of Psychology, University of California-Riverside, Riverside, CA 92521, USA; parke@ucr.edu

**Keywords:** family–peer relationships, parenting, peer relations, child and adolescent development, child and adolescent social development

## Abstract

Nearly thirty years ago, we invited a consortium of esteemed researchers to contribute to a volume entitled *Family–Peer Relations: Modes of Linkage* that provided a state-of-the-science appraisal of theory and research within the newly emerging discipline of family–peer relations. The volume’s first chapter was titled, “Themes and Theories: Perspectives on Processes in Family–Peer Relationships”, and its primary aims were to identify the processes in the family system that were posited to have a bearing on children’s development in the peer system (and vice versa), characterize potential mechanisms of linkage, describe extant lines of investigation, appraise empirical accomplishments, and identify issues in need of further investigation. Here, nearly thirty years hence, we are pleased to have the opportunity to reappraise the theory and research on family–peer relations. In this article, we revisit the primary objectives that were addressed in our previously published “Themes and Theories” chapter but do so with the express purpose of evaluating the discipline’s progress. Likewise, we also revisit our prior roadmap and associated calls-to-action to update these entities in light of past accomplishments, current limitations, and pressing sociocultural issues and concerns.

## 1. Themes and Theories Revisited: Perspectives on Processes in Family–Peer Relationships

Nearly thirty years ago, we invited a consortium of esteemed researchers to contribute to a volume entitled, *Family–Peer Relations: Modes of Linkage* [1]. Our goal for that volume was to provide a state-of-the-science appraisal of theory and research within the newly emerging discipline of family–peer relations. The volume’s first chapter was titled, “Themes and Theories: Perspectives on Processes in Family–Peer Relationships”, and its primary aims were to identify the processes in the family system that were posited to have a bearing on children’s development in the peer system (and vice versa), characterize potential mechanisms of linkage (e.g., cross-system mediators), describe extant lines of investigation, and appraise empirical accomplishments. A secondary aim for the chapter was to construct a roadmap of essential new research domains and focus attention (i.e., via calls-for-action) on issues in need of further investigation (i.e., theoretical controversies, understudied hypotheses, etc.).

Now, nearly thirty years hence, we are pleased to have the opportunity to reappraise the theory and research on family–peer relations. In this article, we revisit the primary objectives that were addressed in our previously published “Themes and Theories” chapter but do so with the express purpose of evaluating the discipline’s progress. Likewise, we also revisit our prior roadmap and associated calls-to-action to update these entities in light of past accomplishments, current limitations, and pressing sociocultural issues and concerns. 

## 2. Premises Undergirding Theory and Research on Family–Peer Relations

Theory and research on family–peer relations have been predicated upon multiple, foundational assumptions and premises. The most basic of these is that families constitute a primary socialization context in which participants (e.g., parents) instigate and engage in processes that impact children’s social competencies (or lack of them) and thereby their relations with peers. A related assumption is that individual differences in children themselves are responsible for variations in their social competencies and for the extent to which those competencies are malleable or responsive to familial socialization. In addition, the premise that specific modes of linkage exist between the family and peer systems, such that developments that occur in one context (e.g., growth of a child’s competencies) transfer or influence developments that occur in the other context, is also central. 

Premises about the mechanisms that account for these linkages (e.g., mediating processes) vary across theories, but it is generally assumed that multiple pathways exist and that the direction of the effects of these cross-system linkages not only are unidirectional, but also bidirectional or transactional (from the family to the peer system and vice versa). Foremost among the mediating processes postulated thus far are parents’ and children’s encoding and decoding of emotions [2], children’s internal working models of relationships [3], children’s emotional regulation abilities [2,4], children’s social information processing [5], and children’s attentional and self-regulation abilities and difficulties [6].

## 3. Overarching Theoretical Perspectives and Controversies

The investigation of family–peer relations, which began in earnest during the 1980s, was prompted by a confluence of conceptual forces. Particularly influential were tenets from ecological theory, which held that the family and the peer systems operate as interconnected contexts within a broader, multilayered social milieu [7]. Another principal impetus was a research initiative implemented by peer relations researchers that was based on the assumption that children’s social competence originated within the family [8]. 

In early studies, investigators formulated hypotheses about family influences on children’s peer relations and sought to substantiate these linkages by examining the associations between family-specific attributes or processes (e.g., parent–child attachment) and qualities of children’s peer relations (e.g., friendship quality). The inquiries eventually shifted toward more complex aims, such as elucidating the processes of relationship learning, the transfer of such learning across contexts, variations attributable to individual differences in children, and the conditional nature of parent–child connections and their effects on child outcomes [9]. Movement in these directions was accompanied by the emergence of alternative, potentially competing, explanatory paradigms, and these frameworks altered investigators’ agendas and challenged assumptions about the mechanisms underlying the links between the family and peer systems. Two overarching theoretical perspectives, in particular, sparked debate and shaped subsequent research agendas. 

The first, and predominant of the two paradigms, was anchored in environmental-organismic perspectives (e.g., social learning theory) and emphasized socialization in the family as the means through which children acquire social competencies that ultimately carried over into their relations with peers. Although this paradigm and its underlying premises have evolved to incorporate theoretical revisions and changing conceptions of causality, a basic tenet has been that parents influence children’s social development. For this reason, researchers who work in this tradition have been interested in explicating family processes as determinants of children’s success or difficulty in peer relations. 

The second, later-emerging paradigm, was founded on the principles of behavior genetics and raised doubts about family processes as a formative influence on children’s social skills and peer competence [10]. Researchers who embraced this perspective argued that the parents’ and child’s shared gene pool, and its interaction with rearing experiences (e.g., shared/nonshared family environments), were responsible for children’s sociability and its consequences (e.g., a child’s behavior and associated relational outcomes) in both the family and peer contexts [11].

## 4. Conceptualizing Pathways between the Family and Peer Systems

A key theoretical and investigative challenge for researchers has been to identify and differentiate among the types of pathways, or modes of influence, that account for cross-system changes in children’s development. In research on family socialization, the following two types of processes have been proposed and investigated as potential pathways of influence: (1) those that occur as part of family life, and most likely derive from relationships and dynamics that are internal to the family system (rather than external to it, such as within the child’s peer environment) and (2) those that transpire in the family context or with family members, but are predicated on children’s actual or anticipated experiences in the peer milieu, or parents’ perceptions of a child’s needs within the social context [1,12].

For organizational and heuristic purposes, the mechanisms included in the former category are termed *indirect* because they refer to aspects of family life that may affect children’s social competence but represent modes of influence that do not provide a child with an explicit connection to the world of peers. In contrast, *direct* modes of influence encompass parents’ efforts to socialize or “manage” children’s social development, especially as parenting behaviors and strategies pertain to the peer context. Mediating variables, such as learning experiences that children acquire in the family that transfer to the peer context, are also considered.

Distinguishing between indirect and direct family influences does not, however, preclude the possibility that both forms of socialization operate simultaneously within children’s rearing environments and have combined effects on children’s competence with peers. Nor should this conceptualization imply that the direction of the effects of these cross-system linkages is exclusively unidirectional (e.g., always from family to the peer system). Although most research conducted thus far has been based on “family effects” models, it is probable that peer system processes precipitate changes or affect children’s development within the family system.

## 5. Overview of Investigative Avenues and Advances/Accomplishments

Since the discipline’s inception, researchers have investigated numerous aspects of the family and, in the majority of these studies, have worked from a family-effects perspective. The resultant body of findings, in large part, was derived from two principal avenues of investigation: one that examined indirect family influences and another that explored direct family influences.

As comprehensive reviews of these findings have been published recently [12,13], our purpose in the ensuing paragraphs is to (1) summarize the key perspectives and premises that have guided past and present research, and (2) briefly illustrate exemplary historical and modern research trends (i.e., foundational vs. recent aims, findings). Subsequently, we also consider alternative investigative avenues and innovations, and identify topics and issues that warrant further investigation.

## 6. Research on Indirect Family Influences

Studies of indirect influences, or family processes and dynamics that are not integral to peer socialization, were among the first to be implemented in the family–peer relations discipline. Focal dimensions included parent–child attachment, childrearing styles, parenting behavior and parent–child interaction, parents’ attitudes and beliefs, parental discipline and stress, marital discord and divorce, family pathology, and child abuse.

### 6.1. Parent–Child Attachment

Parent–child attachment figured prominently in the study of family–peer relations because theorists [14] maintained that early attachment relationships laid a foundation for children’s emotional and psychological development (i.e., emotional security vs. insecurity; autonomy vs. dependence or resistance; adaptive vs. dysfunctional internal working models of relationships [3,14]) that was essential for the establishment and maintenance of future relationships, including those with peers.

Early studies largely supported the premise that children’s early attachment status was a precursor of their subsequent interactions and relationships with peers. Investigators found that securely attached preschoolers, as compared to their insecurely attached counterparts, were more sociable and cooperative and formed better peer relationships [15].

Evidence from contemporary studies and with older age groups further substantiated and extended these inferences. For example, meta-analyses of findings from numerous studies conducted over substantial time periods suggested that (1) along with other family factors, attachment status was a significant predictor of children’s success in peer relations, particularly friendships [16], and (2) attachment security was more closely linked with positive features of children’s peer relationships than was attachment insecurity, and all forms of insecure attachment (i.e., insecure subtypes) were associated with lesser competence in peer relations [17,18].

The evidence linking secure attachment status with later peer competence and relationships now extends beyond preschool into middle childhood, adolescence, and adulthood [19,20]. These findings imply that the benefits of early attachment security persist across the lifespan [21]. The premise that such outcomes are mediated by children’s internal working models of relationships [3] has been corroborated [18,19], but requires further substantiation. The hypothesis that child characteristics, such as temperament, underlie, or account for, the relations observed between attachment and children’s peer relations has not received strong empirical support [17,18]. Father–child attachment has not been studied as extensively as mother–child attachment, and its contributions to children’s social competence have not been fully explicated. More has been learned about the mediators and moderators of attachment status and its links with children’s peer relations. In particular, the hypothesis that attachment is of greater consequence for boys’ than girls’ social development [22] has not been supported by meta-analytic findings [16,18]. Likewise, there has not been strong support for the premise that individual risk factors (e.g., poverty, fetal exposure to teratogens, child psychopathology), strengthen or weaken the effects of early attachment security on children’s social development [17,18]. Some evidence, however, suggests that multiple risks disrupt the linkage between attachment and peer competence [23].

Overall, attachment research has advanced substantially. Many of the issues, limitations, and recommendations raised in our 1992 chapter (e.g., the need to examine attachment’s impact beyond early childhood, the significance of working models, the role of temperament, the relevance of father–child and other nonmaternal attachments) have been addressed, although some have been researched less extensively than others, and numerous challenges remain.

### 6.2. Childrearing Styles

While attachment theory focuses primarily on emotional and relational developments that transpire between parent and child within a circumscribed, sensitive period in a child’s development (i.e., infancy, toddler years), the frameworks that guide research on parenting styles posit modes of influence that endure across development and, in some formulations, are transformed over time to fit a child’s level of maturity.

Early research on childrearing styles was based on the assumption that differences in parents’ warmth and control, as characterized within specific typologies (i.e., authoritarian, authoritative, permissive, and indifferent–uninvolved parenting styles) were influential in shaping children’s peer competence and relationships. It was proposed that childrearing styles differentially impacted children’s social skills, through modeling and other social-emotional learning processes [24,25], and that children transferred these skills to the peer context.

Initial findings included evidence indicating that authoritarian parenting was associated with children’s social incompetence and peer-related difficulties (e.g., inability to initiate positive interactions, hostility, and decreased empathy [26]). In contrast, authoritative parenting was predictive of multiple facets of children’s social competence (e.g., interpersonally maturity, assertiveness, confidence in social interactions [27]).

In contemporary studies, researchers studied parenting styles by measuring arrays of parenting characteristics that corresponded to individual typologies (e.g., supportiveness, warmth, closeness, age-appropriate maturity demands for the authoritative style vs. hostility, coerciveness, dominance for the authoritarian style [28]). In these studies, results showed that characteristics of authoritative parenting were positively associated with children’s social competence, whereas dimensions of authoritarian parenting—and to a lesser extent permissive parenting—were linked with children’s aggressive and disruptive behaviors among peers [29].

In general, although researchers continue to study childrearing styles, mounting evidence suggests that parenting typologies are not as robust as originally conceptualized [30]. Moreover, it has been argued that such typologies constitute trait-like conceptualizations and imply that parents utilize the same styles across all interactions and contexts, regardless of changes in children. As such, this dimensional approach fails to recognize bidirectional influences between parents and children. Another limitation is that parenting typologies may have different meanings both across ethnic groups in North America and cross-culturally [31,32] and, thus, may be linked with culture-specific aspects of children’s peer competence or difficulties. For these and other reasons [12], many researchers have chosen to investigate the role of parenting behaviors rather than typologies in the development of children’s peer relations.

### 6.3. Parenting Behaviors

The movement to investigate specific parenting behaviors largely evolved from earlier research on parenting styles. For example, investigators found that behaviors indicative of authoritarian parenting (e.g., harsh control, coerciveness, directiveness, hostility/rejection, intrusiveness) were associated with children’s lack of social competence, aggressive tendencies, and a greater risk of bullying and peer victimization [32,33]. Alternatively, parent behaviors indicative of authoritative parenting (e.g., warmth, responsiveness) were associated with children’s success at forming positive peer relationships [34,35,36] and specific social skills, such as emotional expressiveness and control [37].

More recent evidence suggests that parental support may moderate the links between the behavior problems of best friends. As Havewala et al. (this issue) found, the internalizing problems of a best friend predict an adolescent’s externalizing problems only at low levels of maternal and paternal support. Clearly, the impact of friends on each other’s behavioral problems can only be fully understood in the context of parent–child relationships. 

Other parenting behaviors linked to children’s peer relations included parental negativity and psychological control. For example, Rispoli et al. [36] found that observed parental negativity when interacting with their children at two years old was directly related to poorer socioemotional skills and behavioral functioning when their children entered kindergarten. Further, this link was mediated by increases in children’s negativity. In research with adolescents, Cook et al. [38] found that parental psychological control was associated with adolescents’ later friendship difficulties.

Thus, in contrast to global typologies, research on specific parenting behaviors tends to reveal more nuanced links with children’s social competence. For example, researchers have found that specific negative parenting behaviors differentially predict boys’ and girls’ risk of peer victimization such that, coercive emotional control (intrusiveness) and lack of responsiveness are correlated with peer victimization in girls, but maternal overprotectiveness is associated with peer victimization among boys [39]. Moreover, when mothers’ and fathers’ negative parenting have been compared, results suggest that fathers’ harshness has a stronger influence on children’s aggressiveness toward peers than mothers’ harshness does [40]. 

### 6.4. Parent–Child Interactions

Rather than examine the parents’ behavior or rearing styles, another investigative approach was to observe parents’ and children’s interactions. In early studies, researchers investigated parent–child play and discovered that certain parental activities or roles during interactions (e.g., directiveness, sustaining children’s interactions, regulating children’s affect, etc.) were linked with the quality of children’s peer relations [41]. Other interactional processes found to predict children’s competence with peers included parental modeling and response evocation [25]. 

Subsequent trends included expanding the investigation of parent–child play as well as other formative contexts, such as parent–child conflict and emotional socialization (e.g., expression, discussion of emotions). Research on parent–child conflict, for example, showed that the mother’s styles of arguing were linked with the children’s strategies for handling conflicts with friends [42]. Other studies revealed that mothers’ negative overtures and maternal and paternal disconfirming behavior correlated negatively with children’s prosocial behavior, and that disconfirming behavior correlated positively with children’s aggression [43]. Studies of affective exchanges and discussions suggest that parents’ contributions to emotional socialization carry over into children’s peer relations. Much of this work has been based on the premise that parents’ responsiveness to children’s distress provides behavioral models as well as opportunities for children to practice managing their own and others’ distress [37]. Support for this contention comes from evidence indicating that parents’ responsiveness to children’s distress correlates positively with children’s social competence [44]. Other findings point to the importance of parents’ affective expressions during parent–child interactions. For example, investigators found that mothers who often expressed positive affect tended to have socially competent children [45], whereas the reverse was found for mothers who frequently exhibited negative affect [46]. As Lindsey (this issue) suggests, both maternal and paternal emotional regulatory strategies merit attention. When two aspects of emotional regulation, namely the degree of affective suppression and cognitive reappraisal of emotional states were higher with parents, their externalizing behavior in peer contexts was lower. Finally, Jespersen et al. (this issue) expand our ways of conceptualizing how parents, as well as peers, serve as socializers of children’s emotions by their theoretically rich extension of Gottman’s classic work on parental emotional socialization typologies. Not only does this Parent and Peer emotional Responsivity styles (PPERS) framework offer a novel theoretical guide for future work concerning the myriad of ways that parents socialize children’s emotions, but it emphasizes the importance that peers play in the socialization of children’s emotions. Most critically for furthering our understanding of family–peer relationships are the suggestive pathways by which family and peers each contribute to both emotional socialization and to peer competence.

Although we have traditionally viewed parents as the major socialization agents of children’s emotions, it would be valuable to examine the reciprocal influences of emotional socialization strategies encountered in peer contexts on the parental management of emotions as well.

Another trend has been to investigate dyadic qualities of parent–child interaction, such as connectedness, synchrony, and mutual responsiveness, which derive from the theory and research on relationship formation (e.g., attachment theory). In general, findings have shown that children from families where parent–child interactions were characterized by connectedness [34], synchrony [47], or mutual responsiveness [48] tended to exhibit greater competence among peers. 

Recent developments include investigating parent–child interactions and children’s social competence as bidirectional and dynamic systems that, ultimately, shape the competencies that children apply in their peer relations. For example, research suggests that parents respond to shy children by shielding them from threat or distress [49], which, in turn, encourages shy children to avoid or withdraw from challenging social situations, including those that may occur with peers. Additional examples include findings showing that sensitive parenting not only promotes children’s prosocial competence [37], but kind, compassionate, and helpful children tend to evoke more sensitive and warm parenting [50]. Conversely, harsh parent–child interactions appear to promote children’s externalizing behaviors, which, in turn, elicit even harsher parenting [51].

In sum, similar to research on parenting behavior, the study of parent–child interactions departed from frameworks in which parenting influences were construed as broad, trait-like typologies (e.g., stable parenting styles). By targeting interactions, it was possible for researchers to observe individual interaction partners (i.e., the parent or the child), or study characteristics of the dyad (e.g., synchrony, mutual responsiveness). This approach was also better suited to analyzing and disentangling the sources and directions of effect (e.g., partner effects, bidirectional influences). Thus, this avenue of investigation broadened the unit of analysis to include more of the family context (e.g., the parent–child dyad) and expanded the purview of potential cross-system modes of influence. 

### 6.5. Parental Discipline

In research on family–peer relations, investigators have primarily examined assertive and inductive discipline. While assertive discipline entails the use of verbal commands and physical power (e.g., corporal punishment) to discourage unwanted behavior [52], inductive discipline relies on reasoning (e.g., instructions, rationales, projected consequences) to encourage desirable behavior [53]. In theory, assertive discipline is likely to promote power-assertive and aggressive behavior in children because it models similar tactics (e.g., hitting/spanking, yelling), and fails to convey rationales that discourage wrongdoing [54].

Early studies revealed that children exposed to assertive discipline displayed aggressive and domineering behaviors, whereas those reared with inductive discipline exhibited prosocial orientations [55]. Other findings linked parents’ harsh disciplinary styles with children’s lack of social skills, aggression, and rejection by peers [56]. Further, Hart et al. [57] found that children whose mothers favored assertive discipline focused on instrumental rather than relational consequences and were disliked by peers.

Subsequent studies suggested that assertive or harsh discipline encouraged children to develop problem-solving skills that exaggerated perceived threats and motivated aggressive tactics toward peers [58]. Other studies revealed that mothers’ physical discipline predicted children’s aggression, even when mothers also exhibited higher levels of warmth [59]. Overall, the evidence characterizes assertive discipline as facilitating children’s aggression and antisocial behavior. 

In sum, research on parental discipline supports the proposition that children learn antisocial behaviors from assertive disciplinary tactics and coercive parent–child interactions and then generalize these behaviors to their interactions with peers. In contrast, the hypothesis that parents’ inductive disciplinary styles teach children prosocial principles that guide their social behavior has received some support but has not been researched as extensively. 

### 6.6. Parental Stress, Marital Discord, and Divorce

Parental stress, discord, and divorce were examined principally in relation to children’s social difficulties. It was proposed that these family processes and events create stress in a rearing environment, degrade parenting quality, and model maladaptive behaviors, all of which negatively affect children’s social development. 

In foundational studies, parental stressors were linked with a variety of childhood social difficulties including social withdrawal, social incompetence, antisocial behavior, and peer rejection [60]. For example, findings showed that parents who experienced more childrearing hassles tended to have children who exhibited social difficulties [61]. Marital discord, another stressor, was linked with children’s emotional problems (e.g., anger, negative affect) and peer difficulties [62]. Likewise, divorce was linked with children’s psychological ill-health and social difficulties [63]. Boys of divorced parents, in particular, tended to manifest aggressive behavior and were disliked by agemates [64].

Newer, longitudinal data tend to corroborate the premise that interparental conflict causes children to develop externalizing problems that, in turn, lead to peer difficulties [65,66]. In some studies, potential mediating and moderating pathways were also implicated. Among the mediators substantiated were disruptions in parents’ childrearing styles and discipline [67] and reductions in children’s emotional security [68]. These findings contrasted sharply with those obtained in research on positive marital relations. Higher marital quality, for example, has been linked with children’s social competence, lower levels of internalizing problems, and, in particular, prosocial skills [69,70].

Overall, findings have been consistent with the premise that parental stress, discord, and disruption reduce children’s social competence, promote maladaptive behaviors, and interfere with the formation of healthy peer relationships. Advances in theory and additional research will be needed to clarify the mechanisms (i.e., mediators, moderators) that link these family processes with children’s peer behavior and relationships.

### 6.7. Parental Perceptions, Beliefs, and Memories

Research in this domain largely has been based on the premise that parents’ cognitions about children’s social characteristics influence their socialization practices [71]. The likelihood of reciprocal influences was also recognized, as in instances where children’s social difficulties become an impetus for compensatory child-rearing strategies [72,73]. 

Initial investigations showed that parents’ perceptions of the difficulty of interpersonal child-rearing tasks correlated inversely with children’s actual social competence [73]. Examination of attributional processes [74] revealed that parents who perceived interpersonal socialization tasks as difficult (and, typically, had a child experiencing peer difficulties) tended to take responsibility for the arduousness of the task rather than blame the child (i.e., a pattern consistent with a “positivity bias” [75]). 

In studies of parents’ beliefs [76], it was discovered that mothers tended to believe that children’s social skills emerged early (i.e., during early childhood) and had different origins (i.e., inherited vs. learned). Particular maternal cognitions were linked with children’s actual social abilities; for example, mothers who placed greater importance on social skills tended to have children who were more skilled at solving social problems. 

Early research also linked parents’ memories of their own childhood peer experiences with children’s social development and peer relations [77]. Investigators found that mothers who had anxious peer memories, as opposed to positive or negative recollections, were more concerned about guiding their children’s peer experiences and relations. 

In later studies, researchers examined the concurrence in parents’ childrearing beliefs, particularly beliefs about control, and children’s competence with peers [78]. Results showed that greater interparental agreement about child control practices correlated positively with children’s peer competence and acceptance. 

Overall, research in this domain was more vigorous and productive during the early, as compared to the later, decades of this discipline. Continued inquiry, it would appear, is merited given that available evidence lends support to the premise that parents’ perspectives guide their rearing styles and behaviors, which, in turn, shape the quality of children’s peer relations. Further, it seems likely that other relevant aspects of parents’ perspectives (e.g., moral values, goals pertaining to the socialization of prosocial vs. violent, aggressive behavior, etc.) remain relatively unexplored and thus merit attention. 

### 6.8. Family Pathology: Parental Depression and Child Abuse

Another hypothesis that prompted investigation was that certain family dysfunctions degrade parents’ child-rearing in ways that curtail children’s social competence and peer relations. Two types of family pathology have received the most empirical attention: parental depression and child abuse. 

Maternal depression has been studied more extensively than paternal depression, and foundational studies were predicated on the hypothesis that depression impacts a child’s adjustment through genetic or socialized transmission of parents’ negative affect, disrupted attachment relations, and disordered parent–child interactions [79]. Research predicated on these premises revealed that the children of depressed mothers manifested antisocial and asocial tendencies amongst peers [80].

In subsequent longitudinal studies, maternal depression during children’s preschool years was found to predict children’s antisocial and aggressive behavior during their early school years [81,82]. Other, longitudinal cross-system linkages implied that maternal depression interfered with children’s ability to develop social competencies [81] and gain peer acceptance [83]. In a rare instance where maternal and paternal depression were studied, investigators found that maternal depression was linked with peer exclusion for girls but not boys, and paternal depression was negatively associated with boys’ but not girls’ prosocial behavior [67].

Abusive parenting was another form of family pathology postulated to impair children’s peer relations. Initial investigations revealed that maltreated children were often aggressive or withdrawn amongst peers, tended to reject peers’ friendly overtures, and were not well received by playmates [83,84].

Subsequently, investigators tested hypotheses about specific mechanisms that might account for maltreatment’s effects on children’s peer competence and relations. Among those substantiated empirically was the premise that maltreatment causes children to become dysregulated (i.e., hormonally, behaviorally, or emotionally) in ways that impair their relationships with peers [85]. Other investigated premises were that physical abuse lowers children’s self-esteem, which, in turn, motivates compensatory reactions, such as seeking enmeshed ties with friends [86], and abusive parental relationships cause children to develop aberrant “working models” that cause children to mistrust peers and degrade their social competence [87].

In sum, although knowledge about parental depression and child abuse has advanced, more remains to be learned about other forms of dysfunction. Potential modes of transmission, including mediators and moderators, also warrant further study. Biologic agents in particular (e.g., genetics, neurological, endocrine processes) deserve further scrutiny, as has been illustrated by the discoveries achieved in research on child maltreatment.

Collectively, most, if not all, of the broader indirect parenting constructs considered here, including aspects of parent–child attachment, relationships, interactions, dynamics, disruptions, and dysfunctions, have been linked empirically with children’s peer competence or relationships. Although substantial progress has been made toward specifying mediating processes that may be responsible for these cross-system linkages, considerable work remains to explicate the nature of these processes, their interrelations, and directions of effects. As an illustration, knowledge remains limited on how children and youth experience various forms of indirect parental influence (i.e., the meaning of these experiences from a child’s or youth’s point of view), and about how their construals of these experiences are related to features of their peer relations (e.g., motivation to engage with agemates).

## 7. Research on Direct Family Influences

Parents’ efforts to promote, oversee or monitor, and manage children’s peer relations have been conceptualized as “direct” forms of socialization that shape children’s relations with agemates. These modes of family influence have been classified into the following four “roles”: parent as designer, as mediator, as supervisor, and as advisor or consultant [12,88]. Each of these roles is considered here, as is the possibility that parents engage in multiple forms of management simultaneously or contingently.

### 7.1. Parent as Designer

Parents act as designers when they seek to establish, control, or influence the settings in which children meet and interact with peers. Among the ways that parents influence children’s access to peers are through their choice of neighborhoods, childcare and preschool programs, after-school care arrangements, and community activities.

#### 7.1.1. Neighborhoods

Unless dangerous or bereft of families, neighborhoods provide places for children to meet and interact with peers [89]. Initial investigations revealed that children have more peer contacts and larger peer networks when they live in densely populated, as opposed to rural, neighborhoods, and in safer, as compared to dangerous, neighborhoods [89,90].

In recent years, investigators have examined how neighborhood features are related to children’s behavior and peer relations, and whether these linkages are mediated by family processes. Caughy et al. [91], for example, found that adolescents living in impoverished neighborhoods tended to be less socially competent and more aggressive. Adolescents’ aggressiveness, however, was less pronounced when their neighborhoods possessed greater social capital (e.g., protections for youth). Further, family cohesion and maternal nurturance significantly mediated these linkages, suggesting that a neighborhood’s influence stems from its impact on adolescents’ families. Along with impoverishment, dangerous and violent neighborhoods have been consistently associated with children’s aggressive behavior. Children who witness violence in their neighborhoods are more likely to become bullies, develop externalizing problems, and join gangs [92].

#### 7.1.2. Childcare and Preschool

Parents’ choice of childcare or preschool has been linked with young children’s social skills and peer relations. Early research showed that children who attended higher- rather than lower-quality programs developed more sophisticated play skills, greater sociability, and longer-lasting friendships [93].

Modern, longitudinal studies of childcare, such as the NICHD Study for Early Child Care and Youth Development, have both corroborated and qualified these early findings. On the one hand, high-quality care was linked with many positive aspects of children’s social development (e.g., sociability, self-esteem, emotion regulation, prosocial behavior [6,94]), as well as children’s long-term social and emotional adjustment [95]. On the other hand, children who spent more time in childcare (i.e., per day, week, year, etc.) were found to be at risk of disruptive, disobedient, and aggressive behavior [96].

#### 7.1.3. After-School Care Arrangements

School-age children’s participation in after-school care has been linked with peer competence and relationships. Early findings cast doubt on the advisability of self-care [97]. To illustrate, the children that were afforded greater self-care in grades one and three were found to have lower levels of peer competence by grade six [98]. Other findings showed that children’s antisocial behavior increased in self-care arrangements, but decreased in formal, adult-supervised arrangements [99].

Subsequent research both substantiated the hazards of self-care (e.g., greater stress, drug use, antisocial behavior [100]) and corroborated the benefits of adult-supervised after-school programs [101]. Pettit et al. [102], for example, found that adolescents’ unsupervised peer contact was associated with externalizing behavior, particularly when parental monitoring was low and preexisting behavior problems were high. In contrast, Pierce et al. [103] found that the flexibility of after-school programs was positively related to boys’ social skills. These findings suggest that developmentally appropriate, adult-supervised after-school experiences foster children’s social competence.

Given that parents are increasing their utilization of childcare and after-school programs, more needs to be learned about parents’ motivation to enroll their children in these settings, especially to the degree that parents explicitly view these settings as opportunities for children to develop peer relationships. Relatedly the extent to which these programs promote peer relationships needs to be noted and the effects of program involvement on peer competence needs to be assessed. 

#### 7.1.4. Community Activities

Another way that parents may facilitate children’s peer relations is by encouraging them to participate in neighborhood and community settings (e.g., parks, libraries) and activities (e.g., clubs, sports). Evidence of such linkages, particularly for young children, was reported in several early studies [104,105]. Likewise, school-age children’s participation in extracurricular activities (e.g., scouts, sports) was linked with their social adjustment [106]. Contemporary research on this parental role has been limited. In one of the few recent studies, McDowell and Parke [107] examined the number of children’s after-school activities and the frequency of their peer interactions in their neighborhoods and found that a composite of these measures positively predicted children’s social competence one year later.

Overall, available evidence lends support to the contention that parents who “design” children’s physical and social surroundings are creating opportunities for children to meet and engage in constructive activities with agemates. Such activities may become an important staging area for the development of social skills, peer relationships, and interpersonal competence.

### 7.2. Parent as Mediator

Parents function as mediators when they create opportunities for children to meet, interact, and form relationships with peers. Often the mediator role takes the form of helping children contact peers, arrange “play dates”, or gain access to peer activities. 

Initial findings suggested that parents’ initiation of peer contacts helped children to establish consistent play companions, develop harmonious peer relationships, and become liked by classmates [72]. Similarly, parents’ sponsorship of informal playgroups was linked with children’s social competence [108].

In subsequent studies, researchers found that older, as compared to younger, preschoolers initiated more of their play dates and received a larger number of play overtures from peers [109,110]. In families where the parents had scaffolded play dates, the children were more likely to self-initiate play dates [110]. These findings implied that parental mediation help children meet and form relationships with peers.

In sum, more is known about some aspects of parental mediation (e.g., parental initiation of informal peer contacts) than others. Further research is needed to clarify when children profit from this type of parental assistance and how parental mediation affects specific forms of child competence (e.g., prosocial skills, friendship formation).

### 7.3. Parent as Supervisor

Supervision has been defined as parents’ efforts to oversee and regulate children’s peer activities and relationships. The following three forms of parental supervision have been investigated: interactive intervention, directive intervention, and monitoring.

#### 7.3.1. Interactive Intervention

This form of supervision has been defined as a parent’s attempts to proactively supervise children’s peer interactions as participants in a play context [111]. In this role, parents are in a position to “scaffold” skills, such as attending to peers, responding to peers’ overtures, and avoiding conflicts. Initial findings indicated that parents’ interactive interventions facilitated toddler’s competence at initiating and maintaining peer interactions [109,112]. Bhavnagri and Parke [109] found that fathers and mothers were equally effective as supervisors and that toddlers benefited more from parents’ interventions than preschoolers did. Directive intervention. With this form of supervision, parents act as observers rather than participants in children’s peer activities and intervene sporadically. Evidence suggests that parents use this form of supervision to remedy children’s social difficulties [111].

#### 7.3.2. Monitoring

Monitoring has been conceptualized as direct and second-party surveillance of children’s peer activities (e.g., parental observation, talks with teachers [12]) and, with adolescents, as gathering information about peer activities from the adolescent themselves [113]. In early studies, researchers found that lesser maternal monitoring correlated negatively with children’s peer acceptance and conduct problems [114,115] and higher maternal monitoring correlated positively with the quality of children’s friendships, particularly for girls [116].

In later studies, researchers focused primarily on adolescents and found that fluctuations in monitoring were linked with changes in adolescents’ deviant behaviors [117,118]. Tilton-Weaver and Galambos [118], for example, found that parents were more likely to seek information about adolescents’ peer activities when the adolescents had problematic friendships or engaged in deviant behaviors. However, in at least one study, the opposite was reported, that is, investigators found that adolescents were more likely to engage in deviant friendships when their parents rarely monitored their activities [119].

#### 7.3.3. Rules

Parents also use rules to guide or control children’s peer activities and, thus, rules have been construed as an indirect form of parental supervision. Simpkins and Parke [116] found that the mothers that reported more supervision rules (i.e., rules about play permission and whereabouts) had sons who tended to be more prosocial. Peer rules (i.e., prohibiting negative or encouraging positive play behaviors), in contrast, were reported more often by mothers of shy boys and aggressive girls.

In sum, the evidence points to age- and context-related differences in the forms of supervision that parents utilize to manage children’s peer relations. Studies are needed to clarify how parents adjust their supervisory behaviors as children mature, and to explicate which forms of supervision facilitate versus impede children’s peer relations. As in the research on monitoring, the question of whether the impetus for parents’ supervisory behaviors lies with the parent or the child (or both) deserves further empirical scrutiny.

### 7.4. Parent as Advisor and Consultant

Parents act as advisors or consultants when they provide children with guidance about peer relations [88,120]. While advising typically takes the form of providing advice or counsel, consulting refers to problem-solving discussions [121] and tends to be instructional in nature [122]. In theory, parents utilize these forms of guidance to assist children with past, present, or future peer-related issues and problems, and may administer such either when peers are present (i.e., in situ guidance) or absent (i.e., “decontextualized discussions” [111]).

#### 7.4.1. Advisor

Investigators initially examined the impetus for mothers’ advice-giving, and its association with children’s peer relations. Laird et al. [122] found that half of the mothers they sampled advised their child on an every-other-day basis and that most of these exchanges were initiated by the child and occurred between mothers and daughters. Cohen [123], in contrast, found that, for supportive, noninterfering mothers, advising correlated positively with children’s adaptive interpersonal outcomes, but for intrusive or disengaged mothers, it was associated with children’s interpersonal difficulties. 

Mothers’ advisory behavior was also examined in structured peer-play situations. Conversations about children’s emotions and problem-solving were the most common forms of guidance [122] and mothers’ advice varied with their child’s peer group status [124]. Mothers of popular and neglected children, as compared to mothers of peer-rejected children, gave advice that was contingent on their child’s actual play behavior.

Subsequent studies produced divergent results. McDowell et al. [125], for example, found that two features of mothers’ advice-giving, higher quantity and quality, correlated negatively with third-graders’ social competence. The investigators noted that advice can be administered both proactively and reactively and concluded that their findings fit the latter direction of effect. In a second study conducted with both mothers and fathers, McDowell and Parke [107] obtained similar results with fourth-grade children.

With adolescents, researchers examined youths’ perceptions of their parent’s advice-giving and studied the role of guidance in the selection and maintenance of peer associates. Mounts [126], for example, found that parental guidance was associated with positive qualities of adolescents’ friendships, and, in a subsequent study [121], reported that this form of parental guidance was associated with adolescents’ assertiveness within peer relationships.

#### 7.4.2. Consultant

Parental consulting was initially studied with children and adolescents. Mize and Pettit [127,128] found that mothers’ social “coaching” (e.g., deliberating with children about social events and potential responses) correlated positively with preschoolers’ peer competence and acceptance. With adolescents, Vernberg et al. [129] found that parents’ consultations about friendship-making predicted adolescents’ successes at making new friends and developing specific friendship features (e.g., intimacy).

In subsequent studies, researchers focused primarily on parents’ consulting practices with adolescents. Consistent with Vernberg et al. [129], Mounts [130] found that parental consulting was related to the quality of adolescents’ friendships. Tilton-Weaver and Galambos [118] examined parents’ peer-related communications with adolescents (i.e., communicating preferences, disapproval, supporting friendships) and found that parents tended to inquire about peer activities and express disapproval when adolescents engaged in deviant friendships.

The confluence of direct parental influences. It has been argued that parents use a range of direct parenting processes to facilitate children’s peer relationships [129], but this premise remains understudied. Exceptions include studies by Mounts [121,130] and McDowell and Parke [107].

In a study of ninth-graders, Mounts [130] examined three parental peer management strategies, conceptualized as guiding, monitoring, and prohibition, and found that guiding was more associated with adaptive adolescent outcomes (e.g., lower friend delinquency and drug use, higher GPAs) than prohibition was. In a second study with seventh-graders, Mounts [121] examined parental guiding and consulting and found that greater parental guidance was associated with adolescents’ assertiveness within peer relationships.

McDowell and Parke [107] studied parents’ advice-giving and provision of peer interaction opportunities with fourth graders and found that both forms of peer management were independently related to children’s social competence. These findings implied that the parents engaged in two forms of peer management simultaneously and that each type of management was positively and distinctly associated with children’s social competence.

In sum, much remains to be learned about direct parental influences. We currently know too little about (1) the extent to which these parental roles occur as antecedents versus consequences of children’s peer competence, (2) the relative importance of differing direct influences for children’s social development, and (3) the extent to which direct influences co-occur and are contingent on other family processes (e.g., indirect influences) and sociodemographic factors. Further, the possibility that some forms of direct facilitation are better for achieving certain socialization goals than are others (e.g., fostering friendships vs. mitigating peer influence) deserves more investigative attention than it has received. Another shortcoming is that studies of indirect influences have primarily been conducted with mothers, and little is known about fathers’ practices or effects.

## 8. Remaining Issues and Future Trends

A variety of issues merit more attention in future work in this area to more fully appreciate the complexities of family–peer relationships. In the sections that follow, we both identify these issues and consider how research in these domains would further our understanding of the linkages between the family and peer systems.

## 9. The Changing Nature of Families

Although much of prior research has been based on a traditional definition of the family as a nuclear heterosexual two-parent family unit, in the past several decades the diversity of family forms has been increasingly recognized [131]. Instead of the two-parent nuclear family, we now have single-parent families in which either a mother or father is the sole parent, same-gender parent families involving two gay men or two lesbians as the parents, adoptive families, as well as multi-generational families residing together as a family unit. The issue is whether adult socializing agents in these different types of family forms manage the task of promoting children’s peer relations in similar or different ways than parents in traditional nuclear families. Children from single-parent families may, in fact, have more behavior problems, display more aggression, exhibit less impulse control, and, in adolescence, engage in more delinquent behavior [132] and be less popular with their peers [133] than children from two-parent families. However, while considerable progress has been made in describing the child-rearing practices (indirect strategies) of single and intact families, less information is available concerning the direct strategies that these two types of parents use in facilitating and managing their children’s peer relationships. Even less is known about single-father families, a growing family form. However, compared to single mothers, they tend to use a more distributed model of child-rearing in which additional caregivers such as their child’s mother, their parents, babysitters, and childcare centers are used as supplements to their own provision of caregiving [94]. In turn, the total amount of contact with additional adult caregivers is directly linked to the child’s warmth, sociability, and social conformity, characteristics that are likely to increase their success with peers.

As the number of children raised in same-gender parent families is on the rise, it is critical to better understand the effects of these family forms on children’s peer relationships. The evidence concerning the social adjustment of children in same-gender families, especially in the case of lesbian couple families that have been most extensively examined, suggests that there are few differences in peer sociability, peer acceptance, or social competence between the early and middle school children of lesbian and heterosexual mothers [134]. A similar story is found among adolescents [135].

More recently, studies of the social adjustment of children of gay couples have confirmed the findings of the earlier studies of lesbian parent families. In an American comparison of gay, lesbian, and heterosexual adoptive parent families, Farr, Forsell, & Patterson [136] found few differences among the groups in terms of either parenting measures or measures of child adjustment. There were few differences in either behavioral or emotional problems of children in gay father families or the other family types. A related study in the UK [137], found that gay adoptive fathers showed less depression and parenting-related stress, higher levels of warmth and interaction levels, and lower levels of disciplinary aggression than heterosexual parents. In terms of child adjustment, conduct problems were lower for the children in gay and lesbian families than for children in heterosexual families.

Although it is clear that the children in these same-sex parent families develop satisfactory peer relationships, the pathways that account for these outcomes are less well understood. The indirect effects, as expressed by the childrearing practices of parents, regardless of family form, on children’s peer relationships are clear: warm and supportive child rearing is related to positive peer outcomes in same-gender families. However, less is known about the direct managerial strategies of same-gender parents. While mothers in heterosexual families are more likely to be managers of children’s peer contacts than fathers, the identity of the parent in same-gender families who plays this role is less clear. Are biological mothers in lesbian parent families who assume more caregiving duties also more likely to manage children’s peer contacts? It is unclear; therefore, this issue warrants examination. Similarly, in gay parent families where there is often a high degree of overlap in the roles played by the two male parents [138], is this managerial role equally shared, and, if not, what determines which parent assumes this responsibility? Finally, these same-gender parents often rely on other opposite gender adults, such as aunts, sisters, and cousins or female friends in the case of gay families, or brothers, uncles, and male friends in the case of lesbian parent families, as supplementary socialization agents in an effort to balance their children’s exposure to the full range of gender role models. What role do these extra-familial agents play in the socialization of children’s experiences with peers? Clearly, we need to recognize that families may employ a range of other agents beyond parents to achieve their socialization goals. 

## 10. Beyond White, Middle-Class, and Western Families

The family–peers literature needs to be more representative of the changing population, which is multi-ethnic and not merely white–European in its composition [131,139]. More studies of non-white families are needed to correct the current imbalance in our understanding of how family–peer relationships vary as a function of ethnicity. Similarly, more cross-cultural studies of how families influence their children’s peer relationships in non-Western cultures such as China are needed (Xiong et al. [140] as an example).

The work by Sigal et al. (this issue), which highlights the role of both maternal and paternal monitoring on drug use in African American and Latinx youth, is a welcome addition and a step toward increasing the generalizability of prior findings on Euro-American families to these other ethnic groups. The inclusion of a Euro-American sample in their study would have further permitted an examination of the relative cross-ethnic differences. A theoretical review by Cox et al. (this issue) further illuminates the role of a shared language across generations in immigrant families. Their analysis offers a novel approach (shared language erosion theory) and an alternative to acculturation as an explanation for understanding the Immigrant Paradox by suggesting that due to the lack of a shared language between immigrant parents and their children, parental influence and control is weakened, which, in turn, leaves children more susceptible to negative non-family influences, such as delinquent peers. These authors propose a rich research agenda that would lead as a next step in the evaluation of the relative contribution of shared language erosion rather than other aspects of an acculturation-based theoretical explanation, such as values, beliefs, and norms, of the Immigrant Paradox.

Additionally, the work of Assari and collaborators (this issue) further underscores the importance of examining minority group status as a factor in children’s academic and social development. Although these authors did not directly examine children’s peer relationships, the same cognitive skills, such as attention regulation, that underlie reading abilities are also correlates of children’s peer competence. Their work on Marginalization Diminished Returns Theory is a reminder that the contextual disadvantages associated with minority status in our society, such as poorer schools, racial discrimination, and reduced occupational opportunities, may limit the advantages of higher parental educational level for the academic success of their children. Their demonstration that this failure of higher parental educational attainment and SES is positively linked to children’s neuro-cognitive indices among minority, in contrast to white, children is a powerful reminder of the need to enact social policies to correct historically based injustices that may underlie these effects.

However, we need to move beyond a replication of findings across different ethnic groups since family structure and values vary across different ethnic groups and these differences have implications for the nature of family–peer ties. For example, the prevalence of extended family forms in African, Asian, Latinx, and Native American families suggests that a wider range of potential socializing agents, such as grandparents, uncles, and aunts, may influence children’s social ties with peers. Not only do these non-parental players influence children’s peer relationships through their child-rearing strategies, but they function as supervisors and monitors on behalf of children in the community. African American grandparents, as well as great grandparents, serve as protectors of their grandchildren in dangerous neighborhoods [141]. Similarly, African American parents who resided in a poor neighborhood, used extended kin who lived in more affluent neighborhoods as safe havens for their children, and, in turn, to reduce the influence of negative social influences on their children [142]. Community members often form neighborhood watch groups to monitor their children [143], and, as a result, children who live in supportive neighborhoods are better socially adjusted [144]. In Latinx families, not only do extended family members play significant child-rearing and monitoring roles but godparents, or compadres (co-fathers) and comadres (co-mothers), play important roles not only as role models but as supervisors and monitors of children’s activities with peers in the community. Moreover, there are protective effects of extended family arrangements for children, including less deviant behavior [145]. Latinx children and youth are more family-oriented and have higher feelings of respect for family and a higher commitment toward family assistance than children in European American families [146]. Asian American families too are more likely than Caucasian families to reside in extended family households and to place a higher value on family obligations [147]. In both Latinx and Asian families, these family profiles are especially evident in immigrant families [139]. Likewise, Native American, American Indian, and Alaskan Native families also include multiple generations living in multiple households [148] in which the distribution of socializing and supervisory responsibilities is shared across extended family members. However, the specific strategies used by either parents or extended family members in influencing children’s peer relationships have rarely been addressed (for an exception see Mmari et al. [149]).

Finally, the role of siblings in families varies across ethnic groups in Western cultures as well as in non-western cultures. In some groups, such as Latinx American families, older siblings play significant caregiver and supervisory roles. Although siblings can play positive roles as supervisors and protect their younger siblings from negative social influences, they may expose each other to risks, including illicit substance use, delinquent behavior, and association with antisocial peers [150]. Siblings can also aid in deviancy training by serving as antisocial models, reinforcing antisocial behaviors and attitudes, and colluding to undermine parental authority [151]. We need to better understand how siblings alter children’s relationships with their peers, how parental and sibling managerial roles complement each other, and how these sibling influences are enacted in different ethnic groups. The challenge for our field is to examine the specific roles that these variations in family composition and family values across families of different ethnic backgrounds play in accounting for peer outcomes.

## 11. Historical Shifts in Family–Peer Relationships

It is increasingly recognized that secular shifts across historical periods influence the nature of family–peer relationships and question the ahistorical assumption that the same set of principles can adequately account for these cross-social-system ties across different historical eras [152,153]. This viewpoint suggests the need for monitoring changes across time in the nature of family–peer linkages to determine how changing circumstances alter these cross-system ties. Historical variations, such as war, famine, floods, economic downturns, refugee migration, and, most recently, the COVID-19 pandemic, provide important natural experiments that permit opportunities for theory and model evaluation.

During wartime deployment, shifts in parent absence, especially father absence, can allow for the evaluation of the relative importance of fathers in, for example, the management of children’s peer relationships [154]. Another example flows from the shifts toward a market economy in China, which was accompanied by a change in the favored child characteristics from a preference for shyness to assertiveness and self-direction as desirable traits. In turn, there were related shifts in child traits associated with higher peer acceptance [155]. In light of these secular trends, one can ask whether the findings of Hu et al. (this issue) concerning maternal autonomy granting reflect this overall cultural shift toward a self-direction or is a more generalizable finding across the past few decades. One would expect that these shifts in preferred child traits would be accompanied by changes in parental socialization to maximize their children’s peer social status.

Another secular shift that merits more attention is whether family–peer relationship patterns have changed in response to the shifting means of communication available to peers in the era of the internet and cell phones. Parental monitoring of peer contacts in this new communication era may present new challenges. For example, how effectively are parents able to track their children’s internet-based social contacts? Assessments of parental knowledge of their children’s peer contacts and an examination of parental efforts to track and monitor these contacts would be useful as a first step in addressing this issue. Furthermore, an assessment of the impact of parental interventions to regulate these new communication era contact patterns on the nature of these peer contacts and, in turn, on parent–child relationships would be valuable.

This issue of parental management of peer relationships at a distance has been brought into sharp focus as a result of the recent COVID-19 pandemic. Many children were unable to attend school or childcare and, as a result, were deprived of their usual face-to-face social contact with peers [156], which, in turn, was associated with increases in depression and lower levels of life satisfaction [157]. Moreover, because of the restrictions imposed by the need for social distancing and limiting the range of individuals with whom one can safely have face-to-face contact, parents face a serious challenge of aiding their children to maintain social ties with their peers. The extent to which parents have encouraged, facilitated, and regulated their children’s peer contacts through the use of social media in this era of COVID-19 and, in turn, its impact on the quality of children’s peer ties needs to be examined. Finally, it is important to assess the mutual impact of limited peer–peer contact on the quality of parent–child relationships during this pandemic, where parents assume a more intensive and central role in the daily lives of their children. To illustrate, parent–child conflict is a further predictor of poorer mental health outcomes for youth, in part due to lessened peer contact and increased and enforced parent–child contact [157].

## 12. Beyond Single Pathways: The Need for Multiple Pathway Models

Although there is substantial evidence that both indirect and direct family pathways are important determinants of peer outcomes, less is known about the combined impact of these parenting processes on peer relationships. To date, research has largely focused on a solitary or a limited number of processes in a single study and leaves unanswered the issue of how these processes combine to produce their effects. This suggests the need for more designs in which both indirect and direct processes are examined simultaneously in a single study to assess the relative importance of each of these parental strategies on peer outcomes. For example, McDowell and Parke [107] found that each of three forms of parenting (one indirect and two direct processes) were independently (additively) related to children’s social competence, suggesting that not one, but many, parenting processes contribute to children’s social competence. More work involving children at different developmental levels is needed to more fully understand how processes combine and whether different processes vary in importance across development. While parental functions, such as direct supervisor and arranger, may be important for younger children, monitoring may be more critical for adolescents.

Child temperament may play a role in shaping parental strategies. Do extraverted and socially oriented children encourage parents to provide more opportunities for social contact with peers relative to shy children? We know relatively little about the real-life determinants of parental decision-making regarding the strategies that they choose to enact and a roster of descriptive typologies of indirect and direct processes across an array of families would be useful. Moreover, we need to explore whether some combinations are more effective socialization strategies for promoting peer ties than others or whether a variety of combinations can yield positive outcomes in recognition of the fact that successful developmental adaptations can be achieved through multiple combinations of parental influence.

## 13. Developmental Levels Need More Recognition as Determinants of Parental Strategies 

More work involving children at different developmental levels is needed to fully understand how processes combine and whether different processes vary in importance across development. Many of the indirect processes that encompass child-rearing practices are important at all levels of development, although even these practices shift as the child develops and gains more autonomy and independence in adolescence and beyond. Even more development-related shifts occur in the direct parental influences that we noted above. For example, while proximal parental roles, such as direct supervisor and arranger, may be particularly important for younger children, more distal roles, such as monitoring and limit setting, may be critical for adolescents. In addition, as revealed in work on adolescence, parental influence is a negotiated activity between the adolescent and parent rather than a parent-to-child influence process [117].

In the adolescent period, romantic relationships become salient but parental influence continues to be evident in this aspect of peer relationships. Adolescents who have close relationships with their parents have closer romantic relationships and if they can successfully resolve conflicts with their parents they are better able to manage conflict with romantic partners [158]. Although these indirect influences of parent–child relationships on later romantic ties in adolescence are relatively well documented, the direct role of parents in the management of romantic relationships through monitoring and other direct strategies is less well understood. An area in need of more attention is the influence of family on adolescent same-sex romantic relationships. For example, adolescent boys with same-sex attraction rated their relationships with their fathers as less satisfactory than heterosexually attracted boys [159]. The question, “How do levels of parental acceptance or parental disapproval influence the relative success of same-sex romantic ties?” merits closer examination as part of the exploration of family–peer relationships.

The age range in which offspring remain dependent on their parents has expanded in recent decades to include the phase of young adulthood. Therefore, we need to devote more attention to the ways in which parents continue to seek to shape and influence the extra-familial social lives of young adults who are often still co-residing in their family home and who are less likely to be financially independent than in earlier decades [160]. Future studies need to examine parental expectations and practices concerning their roles in regulating the social lives of young adult offspring as well as the perceived legitimacy of such parental control on the part of young adults. Clearly, extending our inquiry of the family–peers issue into young adulthood would be worthwhile. A further descriptive profile of how parental strategies aimed at shaping children’s peer relationships shift across the full span of development would be valuable.

Another issue concerns what combinations of strategies parents choose across development as a function of their ethnicity or the characteristics of the child. For example, Latinx American fathers more closely monitor and are more protective of their adolescent daughters than their sons, while this gender-linked pattern of monitoring is less evident in Euro-American families [161].

## 14. Beyond Single Mediating Process Accounts

As outlined in our review, a number of factors likely operate as mediators between family and peer systems, such as social information processing strategies (i.e., cognitive representations of peer and family relationships, cognitive working models), emotional processes (i.e., emotional encoding and decoding skills, emotional regulation), and attention regulation capacities. However, their relative importance in terms of variance accounted for in our models remains unclear since many studies focus on a single or a limited number of mediators in a single study. More multi-mediator studies are needed to address this issue and to begin to assess the relative importance of these potential mediators and to determine which particular combinations of mediators are most effective as explanatory accounts.

## 15. Biological and Genetic Determinants of Family and Peer Relationships

It is increasingly clear that both genetic and environmental factors merit consideration in our accounts of family–peer relationships. Especially promising as models are recent studies on the combination of gene and environment interactions, which suggest that certain child-linked genetic predispositions are likely to result in poor social outcomes but depend on the child-rearing practices of their parents or environmental stressors. For example, Brody et al. [162] found that children who have a version of a gene (5-HTT) associated with an increased risk of depression, lack of self-control, and drug use, and were reared in a family environment in which parents were uninvolved and unsupportive of their children, were more likely to abuse drugs during adolescence. In contrast, youth with supportive and involved parents, despite their genetic predisposition for drug use, did not show an elevated pattern of drug usage. Similarly, children at a genetic risk of depression are only likely to develop serious depression when they are reared in a stressful family environment, which is another reminder that both genetic and environmental factors merit consideration in our future studies of family–peer relationships [163]. In related work, Caspi et al. [164,165] found that children with the MAOA genotype (a stress regulating gene) who were reared in an abusive family exhibited elevated forms of antisocial behavior and lower mental health. Although there have been many recent studies of G x E interactions [166,167], there is still a healthy debate regarding the replicability and robustness of these findings since the original studies often involved small samples [168]. While suggestive, these studies did not typically include standard measures of peer competence; therefore, conclusions about the role of these G x E factors in accounting for family–peer relationships remain unclear. Future studies would benefit from genetically informed designs in which genetic markers, parental behavior, and peer outcomes are assessed simultaneously in a single design.

## 16. Toward More Complex Cross-Time Designs

A major challenge for this field is to develop and test more complex models that reflect the cross time and transactional nature of the links between family and peer systems. Too often, studies leave open the question of direction of effects and, even more problematically, fail to appreciate the transactional nature of the links between these systems. While the role of parents in shaping the nature of children’s peer relationships, especially among young children, is clear, the bidirectional and transactional characterization of these family–peer links, especially as children develop, need to be more fully recognized. For example, children’s negative experiences at school with peers can alter subsequent parent–child interactions. Children who reported experiencing problems with peers at school were more likely to describe themselves as difficult and demanding with their parents later in the evening [169]. Similarly, Lehman and Repetti [170] found that following a problematic day with peers at school, children reported having more aversive interactions with parents at home. 

The work of Hu et al. (this issue) provides contemporary evidence in support of the bi-directional argument but also illustrates the transactional nature of the family–peer mutual influence process. In this study, not only was parental autonomy predictive of later peer preferences, but healthy peer relationships had a positive effect on maternal autonomy support in girls as well. Further signs of progress toward the embrace and testing of transactional models are evident in work by Gazelle and Cui (this issue) as well. Their findings, that youth with fewer reciprocated friendships predicted increased maternal over-control, which, in turn, predicted a decrease in reciprocated friendships, illustrate not only parental responsivity to children’s relative success with their peers but also clearly the transactional nature of the parent–peer systems across time. Using this work as a guide, the reactive interplay between parents and their children needs to be more fully explored. As Ladd and Kochenderfer-Ladd (12, p. 302) note, “Advances in longitudinal research design and data analyses have made it possible to examine time-varying linkages between variables, alternate across-time variable relations (e.g., cross-lagged contrasts), reciprocation, interdependence among growth trajectories, and other data patterns that may help researchers corroborate or falsify premises about causal relations”.

## 17. Final Reflections

As the articles in this Special Issue and our review have underscored, we have made significant progress in our efforts to demystify the links between family and peer systems. Not only are our samples more representative, but our conceptual framing of the issues has been elevated in its level of sophistication. We now recognize the dynamic transactional nature of these links as a result of our improved designs, more advanced analytic strategies, and our continued commitment to the discovery of mediating and moderating processes that account for these cross-time and cross-system linkages. A continued commitment to unraveling the direction of effects probably needs to include the use of a variety of experimental approaches, such as laboratory, field-based, and theoretically informed intervention studies as well as policy-based natural experiments. Finally, we recognize that both qualitative narrative-based approaches in addition to quantitative designs can together yield a richer portrait of these cross-system relations than either approach alone. In sum, the future of this domain of inquiry looks promising, vibrant, and hopeful, as the papers in this Special Issue so clearly illustrate.
